# Blood heavy metals and brain-derived neurotrophic factor in the first trimester of pregnancy among migrant workers

**DOI:** 10.1371/journal.pone.0218409

**Published:** 2019-06-14

**Authors:** Ye Htet Zaw, Nutta Taneepanichskul

**Affiliations:** College of Public Health Sciences, Chulalongkorn University, Bangkok, Thailand; Chiba Daigaku, JAPAN

## Abstract

**Background:**

Lead, mercury, cadmium and arsenic are the priority heavy metals of major public health concern in industrialized countries. Exposure to them can cause cognitive impairment and depressive disorders through an effect on Brain-derived neurotrophic factor (BDNF) which is an important biomarker of pregnancy. Despite a number of prior studies on heavy metals pollution, there is few of studies on the effect of heavy metals on BDNF during early pregnancy. This study aims to examine the association between maternal blood heavy metals concentrations and BDNF during the first trimester pregnancy among Myanmar migrants in Thailand.

**Methodology:**

This cross sectional study, a part of ongoing birth cohort was conducted at the antenatal care clinic from June to October 2018. A total of 108 with Myanmar migrant pregnancy with a single viable fetus of 0 to 14 gestation weeks who stayed within the industrial plant at least 3 months before were recruited. Socio-demographic characteristics and health behaviors were accessed using a self-report questionnaire. Maternal blood heavy metals (lead (Pb), mercury (Hg), cadmium (Cd) and arsenic (As)) were measured using an inductively coupled plasma mass spectrometer and plasma BDNF was measured using an enzyme-linked immunosorbent assay. Multivariate binary logistic regression were modeled to access the association.

**Results:**

Median (interquartile rank: IQR) concentrations were: BDNF (6.49 (1.79) μg/ml), Pb (2.77 (1.46) μg/dL), Hg (0.62 (0.54) μg/dL), Cd (0.93(0.86) μg/L) and As (0.40 (0.11) μg/dL) respectively. We categorized BDNF concentrations into high (> median) (n = 54) and low (≤ median) (n = 54) groups. After adjusting for potential confounders, high blood total arsenic concentration had 2.6-fold increased odds (aOR = 2.603, 95% CI: 1.178, 5.751) of low plasma BDNF level as compared with low blood total arsenic group. However, there was no significant association between BDNF and Pb, Hg and Cd.

**Conclusions:**

The present findings demonstrate higher blood total arsenic level were more likely to have lower BDNF in early pregnancy. Our study suggested that heavy metal could be worsen BDNF level which plays its important role on biological effect of maternal depressive disorder and newborn neurodevelopment.

## Introduction

More than 25 percent of global burden of disease is related to environmental toxic heavy metals exposure [1]. Heavy metals are systemic toxicants and can induce multiple organs effects even at low exposed levels [2]. In mainly, lead (Pb), mercury (Hg), cadmium (Cd) and arsenic (As) are highly toxic metals. They have been identified as among the priority metals of public health importance by the World Health Organization [3]. Prenatal exposure to these heavy metals is a growing concern because of its adverse effects on the pregnancy resulting an array of negative consequences [4]. The level of heavy metals in maternal blood and its effects on pregnant women has been evaluated in many previous studies. The American College of Obstetricians and Gynecologists recommended that pregnant women with blood Pb level of 5 μg/dL or higher should be identified to receive counselling on prevention of further exposures [5]. In Korea, a recent research pointed out the risk of high Hg exposure as a particular environmental health issue for pregnant women [6]. A study in Japan demonstrated that high maternal blood Cd concentration was significantly associated with negative pregnancy outcomes [7]. A relatively high blood As concentration (median 11 μg/L) was found among Argentinian pregnant women [8]. Heavy metals can enter the human body through skin or inhalation or ingestion of contaminated food or drinking water [9]. Through systemic circulation, they can reach into the brain from the blood by overcoming the blood-brain-barrier [10]. Previous studies have been demonstrated a variety of their toxic effects on pregnancy. But it was noted that the more detailed evidences on their neurotoxicity properties in pregnancy are still limited. In the hippocampus of the brain, they share some common pathways for the mechanism of neuronal cell death and cognitive dysfunction through chemical interactions and oxidative stress. During oxidative stress, heavy metals induce an effect on a particular antioxidant, known as Brain-derived Neurotrophic Factror (BDNF). In addition, BDNF is well known major cellular protective factor in the brain [11]. To the best of our knowledge, there is a little know from previous research investigating the association between heavy metals exposure and the important elements of the brain including BDNF in pregnant women.

BDNF is a member of neurotrophins family which supports differentiation, maturation and survival of neurons [12] [13] [14]. It has a crucial neuroprotection effect for the development and functions of the central nervous system [15]. Additionally, it also plays an important role in the physiology of pregnancy such as follicular development, oocytes maturation, embryo implantation, placentation and fetal maturation [16] [17] [18]. It was found that serum BDNF is significantly low in pregnant women in comparing to non-pregnant women within follicular phase. A recent study reported that decreased serum BDNF concentration in the first trimester of pregnancy was significantly associated with antepartum depressive symptoms. Also, it was recommended that biological assessment of BDNF in early pregnancy is advantageous for risk prediction of antepartum depression [19]. Previously, some studies have examined the mechanism of decreased BDNF concentration during early pregnancy. They described that a number of physiological conditions (hemodilution, fetal sequestration and platelets changes) might be possible factors [20]. Besides, another study pointed out that maternal age and early pregnancy Body Mass Index (BMI) are also significant factors for low early pregnancy BDNF level. Aging and functional alterations of circulating BDNF sources such as neuronal cells, vascular endothelial, smooth muscle cells and activated lymphocytes might be the matter of lowering BDNF level in older age [21]. It was also noted that maternal physical exercise during pregnancy enhances brain development, cognitive functions and BDNF levels of offspring [22] [23] and leads to better clinical outcomes in depressive disorder patients [24]. To date, decreased circulating BDNF concentration during early pregnancy becomes increasingly awakened for maternal health including the offspring with the accumulating evidences of adverse consequences. However, there are still limited studies on the knowledge of BDNF focusing on early pregnancy period. Hence, it is essential to do more research on this issue and it would be very beneficial for the prevention of maternal and offspring's health problems.

Thailand is on a rapidly economic growing progress following a relatively high demand of more industries and greater human workload. There is an increased population of migrant workers from the neighboring countries in which approximately 80 percent are Myanmar [25]. A mixture of industrial and human activities gives rise to higher risk of heavy metals pollution in the environment. Despite some extent evidences of significantly higher levels of heavy metals in Thailand, limited studies had been focused on migrant workers who are a risky group. Together with, there is a limited studies of a link between heavy metal and BDNF. Our study purpose is to study the association between blood heavy metals concentrations and BDNF during early pregnancy among Myanmar migrants in Thailand.

## Materials and methods

### Study design

This study was a part of ongoing prospective birth cohort designed to study the effect of maternal blood heavy metals on pregnancy outcomes among Myanmar migrants in Thailand. Study area was Samutsakhon province which is the second of top ten provinces with largest Myanmar migrants and also a dense of industrial plant with a total of about 6,000 factories manufacturing mainly food and beverage, plastic and plastic products, rubber and rubber products and textiles. A total of pregnant women (n = 108) within 0–14 week's gestation were recruited from the antenatal care (ANC) clinic during June to October of 2018 in a hospital in study area. Eligible criteria were: registered Myanmar migrants with pregnancy visited first time to ANC clinic, age between 18 and 35 years, ability to read, write and communicate Myanmar language, residing in the study area at least 3 months before, planning on delivering at the hospital and willing to give blood samples. Multiparous women with more than 5 parity, less than 1 year inter-pregnancy interval and medical history of depression were excluded. The study was reviewed and approved by the Research Ethics Review Committee for Research Involving Human Research Participants, Health Sciences Group, Chulalongkorn University (RECCU) (COA No.251/2018). Participants were provided with information on the objectives and study design and informed consents were obtained prior to participation.

### Blood heavy metal analysis

A total of 5 ml maternal whole blood was collected in two separate Ethylenediaminetetraacetic acid (EDTA) coated tubes by the trained nurse. One tube (2.5 ml) was to analyze whole blood heavy metals concentrations and another tube (2.5 ml) was to measure plasma BDNF concentration. Samples were sent to standard toxicology laboratory for heavy metal analysis which followed the standard method of ACGIH. Briefly, blood sample was washed in water purification and a 2% nitric acid solution. Digested sample were quantifies using an inductively coupled plasma mass spectrometer (ICP-MS). The detection limits were 0.001 μg/L for all heavy metals. Limits of detections (LOD) were calculated by SD*3.

### Plasma BDNF analysis

Maternal plasma BDNF concentration was measured using the quantative sandwish enzyme-linked immunosorbent assay (ELISA) kit (RayBio, USA) in accordance with manufacturer's protocols. All reagents and samples were bought to room temperature before use. Sample dilution of antibody cocktails and serial dilution for standard solution were prepared according to the procedures. Flat bottom 96-well plates coated with anti-Human BDNF were used to analyze BDNF absorbance. All samples were assayed in duplicate. The immobilized antigen and antibody were incubated with 70 μl of antibody cocktail containing biotinylated anti-Human BDNF and concentrated HRP-conjugated streptavidin for 10 minutes. After incubation, 50 μl of each standard and sample were added into the appropriate wells followed by incubation for 2 hours. The solutions were discarded by adding and washing with 300 μl of wash buffer into 4 times. After washing, 100 μl of tetramethylbenzidine (TMB) one-step substrate reagent was added to each well and incubation at dark room was done for 30 minutes by gentle shaking. Color development was stopped by the addition of 0.2 M sulfuric acid, then the absorbance of 450 nm of each well contents was measured immediately by a Microplate Reader. All incubations were performed under room temperature and it took 2 hours and 40 minutes for total assay time excluding washing process. BDNF concentrations were expressed as μg/ml.

### Questionnaire

After all participants finished the appointment with the obstetrician, the trained research assistant administered the structured self-report questionnaires to the participants which involved socio-demographics characteristics and health behaviors. Participants provided details including age (years), BMI (kg/m^2^), ethnicity (Burma / Others), education (≤ primary school /> primary school), occupation (Employed / Unemployed), monthly family income (THB), duration of residing in the study area (Years), smoking history (Yes/No), secondhand smoke exposure (Yes/No), and behavior of regular aerobic exercises (walking physical activity at least 20 minutes per day) (Yes/No). Content validity of the questionnaire was assessed by three expert committees; one obstetrician, one heavy metals expert and another public health expert.

### Statistical analysis

SPSS software version 22 for Windows (IBM SPSS, version 22, Armonk, NY, USA) was used for analyses. Test of normality was performed using the Shapiro-Wilk test. Both BDNF and heavy metals concentration showed non-normal distributions. Hence, we decided to use binary variables for analysis. The concentrations were categorized into two groups (High and Low) respectively according to their median values. To examine difference between high and low BDNF group on the general characteristics and health behaviors, independent t-test was used for continuous data and Chi-square was for categorical data. Differences of BDNF concentrations between heavy metals groups were analyzed using Mann-Whitney U test. Spearman Rank correlation was used to evaluate the correlation among BDNF, Pb, Hg, Cd and As. Multivariate binary logistic regression models were used to examine associations between BDNF concentration (High: > median, Low: ≤ median) and heavy metals concentrations (High: > median, Low: ≤ median). The models were adjusted for covariate factors including age (years), BMI in early pregnancy (kg/m^2^), history of secondhand smoke exposure (Yes/No) and history of regular aerobic exercises (Yes/No). Potential covariates were selected based on evidences of their statistical significance with BDNF concentration in the previous researches.

## Results

General characteristics of pregnancy among migrant workers are summarized in Table 1. Of a total 108 participants, the median concentration of BDNF was 6.49 (IQR 1.79) μg/ml. Mean age of the high BDNF group was 28.07 (± 4.25) years and of low BDNF group was 28.04 (± 4.07) years. Mean duration of stay in the study area was 5.54 (± 3.10) years for high BDNF group and 5.07 (± 2.61) years for low BDNF group. All of pregnant women in this study reported that they have never smoked. There was no significant difference between groups for age, BMI at early pregnancy, ethnicity, education, occupation, monthly family income, duration of stay in study area, history of secondhand smoke exposure, and history of regular aerobic exercise.

**Table 1:** General characteristics of pregnancy among migrant workers.

Variables	Total (n = 108)	BDNF		p-value
		High (n = 54)	Low (n = 54)	
Age (years)	28.07 ± 4.25	28.11 ± 4.46	28.04 ± 4.07	0.292^a^
BMI (kg/m^2^)	23.84 ± 3.87	23.86 ± 4.03	23.82 ± 3.73	0.549^a^
Monthly income (Thai Baht)	15181 ± 5151.60	15100 ± 5201.05	15300 ± 5148.36	0.687^a^
Duration of stay (years)	5.31 ± 2.86	5.54 ± 3.10	5.07 ± 2.61	0.262^a^
Ethnicity				0.165^b^
Others	62 (57.4)	28 (45.2)	34 (54.8)	
Burma	46 (42.6)	26 (56.5)	20 (43.5)	
Education				0.860^b^
> Primary	62 (57.4)	35 (56.5)	27 (43.5)	
≤ Primary	46 (42.6)	19 (41.3)	27 (58.7)	
Occupation				0.307^b^
Unemployed	19 (17.6)	11 (57.9)	8 (42.1)	
Employed	89 (82.4)	43 (48.3)	46 (51.7)	
Secondhand smoke				0.124^b^
No	53 (49.1)	30 (56.6)	23 (43.4)	
Yes	55 (50.9)	24 (43.6)	31 (56.4)	
Regular exercise				0.624^b^
Yes	85 (78.7)	43 (50.6)	42 (40.4)	
No	23 (21.3)	11 (47.8)	12 (52.2)	

BDNF = Brain-derived neurotrophic factor

^a^ = Independent t test for continuous data

^b^ = Chi-square for categorical data

**Fig 1:**
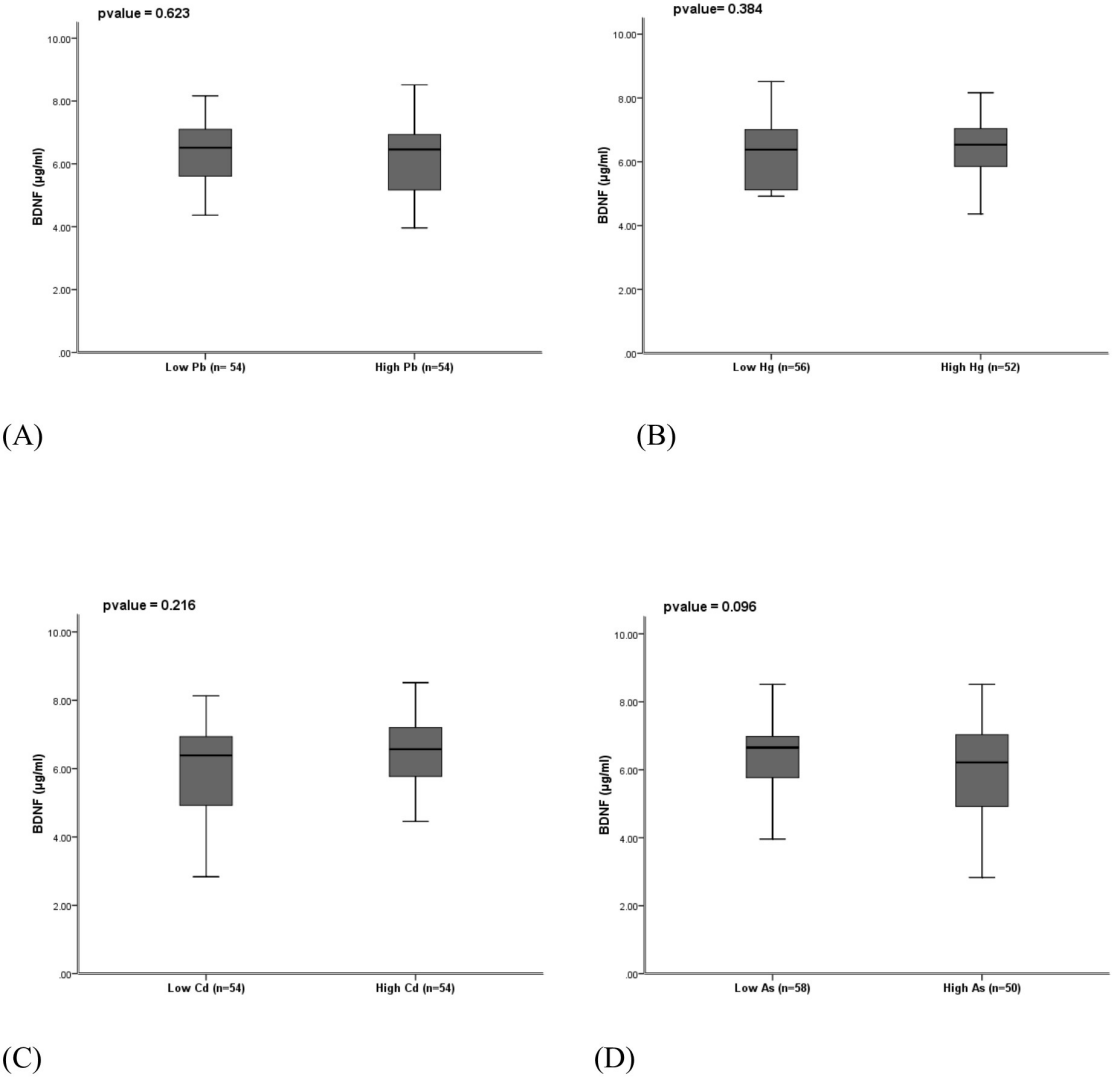
Distribution of BDNF (ng/ml) on low and high blood (a) lead (Pb) (b) mercury (Hg) (c) cadmium (Cd) and (d) arsenic (As) concentrations.

We compared the concentrations of BDNF concentration between high group (> median) and low group (≤ median) of all four heavy metals. Fig 1a shows the distribution of BDNF between high Pb level (> median) and low Pb level (≤ median). The concentration of BDNF is not significantly lower in high Pb group than low Pb group (p-value = 0.623) but minimum concentrations of BDNF were found in high Pb group. Although it was mentioned that there was no significant difference of BDNF concentrations between two groups (p-value = 0.384), the maxium BDNF concentration was found in low total Hg group and the minimum BDNF concentration in high total Hg group (Fig 1b). BDNF concentrations did not differ between high and low Cd (p-value = 0.216) (Fig 1c). For As, there was also no significant difference of BDNF between two groups (p-value = 0.096), however, minimum BDNF concentrations were found in high total As group (Fig 1d).

Table 2 shows blood heavy metals concentration and detection rate. Concentration of heavy metals was measured as μg/dL for Pb, Hg, As and μg/L for Cd. The values of concentrations were calculated in mean ± SD and median (IQR). Total amount of samples was 108 and detection rate was 100% for all heavy metals.

**Table 2:** Blood heavy metals concentration and detection rate.

n = 108	Concentration			Detection rate
	Mean ± SD	Median (IQR)		
Pb	3.10 ± 1.54	2.77 (1.46)	μg/dL	108/108 (100%)
Hg	0.70 ± 0.41	0.62 (0.54)	μg/dL	108/108 (100%)
Cd	0.99 ± 0.48	0.93 (0.86)	μg/L	108/108 (100%)
As	0.41 ± 0.08	0.40 (0.11)	μg/dL	108/108 (100%)

Table 3 shows the correlation between plasma BDNF and whole blood heavy metals including Pb, Hg, Cd and As. There were significant spearman rank correlation coefficients (p < 0.01) between As and Cd, and (p <0.05) between Hg and Cd. Moreover, there was a significant correlation (p < 0.05) between BDNF and Cd but not for Pb, Hg and As.

**Table 3:** Spearman rank correlation analysis among BDNF, Pb, Hg, Cd and As.

	BDNF	Pb	Hg	Cd
Pb	- 0.070			
Hg	0.130	- 0.055		
Cd	0.200*	0.054	0.199*	
As	- 0.127	- 0.094	0.171	- 0.329**

* = Indicates statistical difference, p-value < 0.05

** = Indicates statistical difference, p-value < 0.01

Table 4 shows binary logistic regression models examining the associations between plasma BDNF levels and whole blood heavy metals levels. We performed analyses by categorizing each heavy metals into two groups and BDNF into two groups based on their respective median values. The multivariate model was adjusted for potential confounding factors including age (years), BMI (kg/m^2^), secondhand smoke exposure (Yes/No) and history of regular aerobic exercise (Yes/No). A statistically significant association was found between plasma BDNF level and whole blood total As level in simple bivariate model (aOR = 2.483, 95% CI 1.142, 5.397, p-value = 0.022). Having high blood AS level is increased 2.603 fold-odds of having low BDNF level after adjusting potential confounding factors (aOR = 2.603, 95% CI 1.178, 5.751, p-value = 0.018). There was no significant association between plasma BDNF level and Pb, total Hg and Cd in both regression models.

**Table 4:** Binary logistic regression models of heavy metal and BDNF.

Heavy metals	BDNF		OR	95% CI	aOR	95% CI
	High n (%)	Low n (%)				
Pb						
Low	28 (51.9)	26 (48.1)	1	Reference	1	Reference
High	26 (48.1)	28 (51.9)	1.160	0.545, 2.467	1.230	0.569, 2.660
Hg						
Low	26 (46.4)	30 (53.6)	1	Reference	1	Reference
High	28 (53.8)	24 (46.2)	0.743	0.348, 1.584	0.707	0.324, 1.541
Cd						
Low	25 (46.3)	29 (53.7)	1	Reference	1	Reference
High	29 (53.7)	25 (46.3)	0.743	0.349, 1.583	0.705	0.324, 1.531
As						
Low	35 (60.3)	23 (39.7)	1	Reference	1	Reference
High	19 (38.0)	31 (62.0)	2.483*	1.142, 5.397	2.603*	1.178, 5.751

Adjusted for age (years), BMI (kg/m^2^), secondhand smoke exposure (Yes/ No), regular aerobic exercise (Yes/ No)

OR = Crude Odds Ratio

aOR = Adjusted Odds Ratio

* p-value < 0.05

## Discussion

To our best knowledge, this is the first study on the association between heavy metals and BDNF in early pregnancy. We measured the levels of blood heavy metals and BDNF within early pregnancy of Myanmar migrants in Thailand. Furthermore, we first demonstrated a significant association between whole blood As and plasma BDNF concentration in early pregnancy, but no association between Pb, Hg, Cd and BDNF.

It is well known that BDNF plays a significant role in the pathophysiology of mood and activity of antidepressants [26]. An earlier report demonstrated that circulating BDNF is diagnostic biomarker for major depressive disorders MDD and bipolar disorder (BD) [27]. In pregnant women, a significant association between early pregnancy BDNF and symptoms of antepartum depression was found in the previous studies. They reported that women with BDNF levels in the lowest three quartiles had 1.61 fold greater odds of antepartum depression in comparing to women with BDNF levels in the highest quartile. Therefore, they suggested that early pregnancy BDNF is a potential biomarker to predict antepartum depression [19]. In this study, we can measure plasma BDNF concentration within the first trimester gestation among migrant workers.

Some studies mentioned that normal physiological changes such as hemodilution and platelets changes are likely causes of low BDNF [20]. Probable covariates such as maternal age, body Mass Index (BMI), race and physical exercise were also reported [21]. Furthermore, it was noted that heavy metals have well known neurotoxic effect to induce cognitive dysfunctions. They have an effect on BDNF through an individual or common pathway to produce cognitive dysfunction [28]. In this study, we hypothesized whether the heavy metals are associated with early pregnancy BDNF or not. Although previous studies worked on nonspecific pregnant women, we focused migrant workers working and residing within the probably hazardous area inclusively. We included working-aged, registered Myanmar nationality migrants with no history of depression within their first trimester gestation. We found that about 82 percent of them still attended their industrial occupations during the first trimester. They reported that their average duration of residence within the industrial plant was about 5 years. Although all had no history of smoking, more than half of them informed history of secondhand smoke exposure. Thus, we assumed that the participants were under possible risks of continuously exposure to heavy metals.

For biomarker assessment, we measured blood heavy metals by ICP-MS and BDNF by the ELISA kits. All biomarkers were detectable under the standard procedures. In comparing our results to previous researches, BDNF concentration in our study was much lower than among Peruvian pregnant women [21]. The reason might be genetic, race and physical lifestyle variations. Hence, the more study in other races and detailed comparisons should be needed. Pb concentration in a few of them was higher than the Centre for Disease Control references [29]. Median level for Hg and Cd in our pregnant women were also above standard level for the US general population [30] [31]. Median As concentration was higher than those in the Canadian women [32], but lower than Iranian women results [33]. Those compared studies were not specific to the characteristics of participants. In this study, we focused on migrant population and socio demographic characteristics and health behaviors of them may contribute to some extent higher levels of heavy metals in their early pregnancy. Therefore, our results might be comparable to prior findings and supportive to the existing knowledge.

As is a major environmental toxicant leading to a broad range of health burdens worldwide [34]. As and its compounds have been used for many purposes in different settings such as industrial (alloys manufacturing, pigments manufacturing, electronics, leather preservatives) and agricultural (pesticides, herbicides, insecticides) [35]. Consumption of contaminated drinking water and food are the main routes of As exposure in humans. Also dermal contact to As is considerably important. But, in this study, we did not focus on the specific source and route of exposure. Previous studies demonstrated that As induces multiple disease pathways and chronic exposure causes diverse effects to the central nervous system [36]. The neurological effects of As were most commonly found in the Hippocampus region which plays a major role in carrying environmental signals [37]. By toxicological activity, As exposure firstly produces function alterations in the brain. Secondly, it produces molecules alterations in the hippocampus where it affects BDNF and results cognitive function [38]. It was noted that even its low exposure can induce cognitive dysfunction [39]. One study demonstrated that As has a direct effect on oxidative stress and the imbalances of defensive anti-oxidative mechanism and neurotransmitter metabolism in the hippocampus [40]. In addition, perinatal As exposure has a developmental neurotoxicity with abrupt changes in the reactive oxygen species (ROS), oxidative stress, mitochondrial functions and apoptosis in the developing brain [41].

In this study, we found a significant association between As and BDNF during early pregnancy. In spite of limited researches, our result is consistent to the previous evidences. In the past, many vitro and vivo studies had found that As has an effect to reduce the expression of BDNF significantly. They tested low BDNF expression in the CA1 and dentate gyrus areas of the dorsal hippocampus by administering As contained tap water to mice [38]. Another study proved that As can reduce mitochondira membrane potential (MMP) and decrease BDNF gene expression in As treated human neuroblastoma SH-SY_5_Y cells [42]. As induces hippocampal neuronal apoptosis through an up regulated none morphogenic protein 2 (BMP2), Smad dependent attenuation of BDNF TrkB pathway resulting cognitive impairments [43]. Moreover, it was also found that subchronic As exposure in chemically depression induced mouse model enhances the depression-like behaviors through the cerebral prefrontal cortex BDNF-TrkB signaling pathway [44]. Taken together, the above findings confirmed that As and BDNF are significantly associated on the basis of neurotoxicity and cognitive impairment mechanisms.

The relationship between As exposure and depression among women was discussed in the previous studies. They found that chronic low level As exposure has a positive association with the prevalence of depression and neurobehavioral symptoms among child-bearing aged Indian women. They assessed As exposure level by analyzing contaminated groundwater samples and depressive symptoms by the subjective symptoms questionnaire [45]. Lately a cohort study also indicated that there was an association between low level As exposure in pregnancy and postpartum depression symptoms among Chilean women without any history of depression. They measured As concentration in urine samples and used the standard Edinburgh Postpartum Depression Scale to estimate depressive symptoms [46]. These accumulated findings suggested that As exposure in pregnancy is considerably important for increasing burden of depression in women. In this study, we measured As exposure in whole blood, BDNF in plasma and found a significant association between them. Therefore, our results might be a support to the existing knowledge. But we did not assess the depressive symptoms in our participants and it is needed to consider in the future study.

Considering other metals (Pb, Hg, Cd), evidences of their role for BDNF were found. For example, Pb exposure in rats causes persistent alteration of BDNF expression leading to long term potentiation dysfunction and impaired neuronal plasticity [47]. Cd can compel down regulation of BDNF in the mechanism of neuronal cell death [48]. Besides individual effects, they also have a mixture mode of neurotoxicity in the hippocampus region of the brain. The mixture has a common effect through BDNF which produces more strength to cause cognitive dysfunction. In the hippocampus, they occur dynamic interaction with neurochemicals (e.g. N-methyl-D-aspartate NMDA, acetylcholine esterase AchE, Calcium ions Ca). Then, they cause poor neuronal cell integrity by down-regulation of BDNF and other antioxidants (e.g. catalase, superoxide dismutase SOD). This is followed by the imbalance between the defensive elements and reactive oxygen species (ROS) known as oxidative stress. And it leads to neuronal cell death and ultimately, cognitive dysfunction [26] [49] [50] [28] [51]. In addition, a very recent study found that Pb exposure was negatively associated with serum BDNF concentration among preschool Chinese children. Although they also assessed other metals including Hg, they found no association with BDNF [52]. Nevertheless, in this migrant pregnant women study, we found no significant association between of Pb, Hg, Cd and BDNF during first trimester. Our findings on Pb, Hg and Cd are inconsistent with previous experimental evidences. It would be better to give reason as this is the first human study to examine the association between heavy metals and BDNF in early pregnancy. Therefore, further studies using larger sample size should be needed to confirm.

There are some limitations in this study which might affect the results. Small sample size of Myanmar migrants with averagely similar socio demographic characteristics can limit the generalizability to other population. However, we were still able to examine the significant association for BDNF between blood As in both simple and multivariate binary logistic regressions. Our study did not consider the genetic variations within the participants which might be an effect on metals absorption and distribution. The source of heavy metals exposure was also unspecified in our study which should be considered in the future. We used self-report questionnaire to estimate the behaviors of regular aerobic exercise and it may account for one of our weaknesses. Future studies where participants follow up should be carried out by serial collection of bio-specimens for heavy metals and BDNF across the pregnancy as well as more information on covariates. In addition, we measured BDNF by the ELISA kits which can recognize both mature BDNF and its precursor proBDNF under specificity of antibody. We are not able to differentiate those two BDNF expressions in this study. And a previous study suggested differential diagnostic properties of BDNF expression that mature BDNF is representative for depressive disorders and proBDNF is for bipolar diseases [27] [53]. It would be better to use the advanced ELSIA kits which can differentiate between mature BDNF and proBDNF specifically.

## Conclusion

Our results indicate that plasma BDNF was associated with whole blood As during early pregnancy but not for Pb, Hg and Cd among Myanmar migrants in Thailand. Therefore, As should be counted as an important risk factor for decreased early pregnancy BDNF level and risk of depressive disorders in pregnant women. Future large-scale prospective cohorts are recommended to confirm our findings and investigate the effects of As on BDNF throughout pregnancy, postpartum and among the offspring as well.

## Supporting information

S1 File Content validity of questionnaire. (PDF)

S2 File Self-report questionnaire in English. (PDF)

S3 File Self-report questionnaire in Myanmar. (PDF)

S4 File Data set. (XLS)
