# Supplementary material for: Blood heavy metals and brain-derived neurotrophic factor in the first trimester of pregnancy among migrant workers
Source: PLoS One. 2019 Jun 14;14(6):e0218409. doi: 10.1371/journal.pone.0218409 (PMC6570031; doi:10.1371/journal.pone.0218409)
Supplement: S1 File — (PDF) [file pone.0218409.s001.pdf]

Content validity of the self-report questionnaire

Title

Blood heavy metals and brain-derived neurotrophic factor in the first trimester pregnancy among migrant workers

Researcher

Ye Htet Zaw, MBBS, MMed Sc (Environmental Health), PhD student, Public Health Program, College of Public Health Sciences, Chulalongkorn University

Objective

To examine the association between blood heavy metals and BDNF concentrations in the first trimester pregnancy among migrant workers in Thailand

Self-report questionnaire

It consisted of 11 items related to socio demographic characteristics and health behaviors of the participants. The Item-Objective Congruence Index (IOC) was analyzed for questionnaires' content validity testing by the following expert committees:

1) **Expert (Obstetrics and Gynecology)**
   Dr. May Thu Htun, MBBS, MMed Sc
   Senior Assistant Surgeon, Obstetrics and Gynecology Specialist
   Central Women Hospital, Yangon, Myanmar
   Email: drmaythutun.mc@gmail.com
2) **Expert (Heavy metals pollution)**
   Dr. Pokkate Wongsasuluk, PhD
   College of Public Health Sciences, Chulalongkorn University
   Email: pokkate.w@chula.ac.th
3) **Expert (Public Health)**
   Dr. Wandee Sirichokchatchawan, PhD
   College of Public Health Sciences, Chulalongkorn University
   Email: wandee.s@chula.ac.th

The IOC of the questionnaires was 0.87.

According to 3 experts, the content validity of the self-report questionnaires for this study is approved for measuring.
