# Supplementary material for: Blood heavy metals and brain-derived neurotrophic factor in the first trimester of pregnancy among migrant workers
Source: PLoS One. 2019 Jun 14;14(6):e0218409. doi: 10.1371/journal.pone.0218409 (PMC6570031; doi:10.1371/journal.pone.0218409)
Supplement: S2 File — (PDF) [file pone.0218409.s002.pdf]

Self-report questionnaire

This research is about "Blood heavy metals and brain-derived neurotrophic factor in the first trimester of pregnancy among migrant workers". You are kindly asked to answer the following questions yourself. Please answer the questions by placing an "X" mark over the box next to the response that you have chosen. The questions in this part contain those dealing with your socio demographic characteristics and health behaviors. As with any part of the questions, you are free to choose not to participate or if you choose to participate, you can skip any question that you do not wish to answer. Your responses are confidential as neither your name, address nor any other identifier will be attached to any of your responses.

1. Age

   .... (years)

2. Weight

   .... (kg)

3. Height

   .... (cm)

4. What is your average monthly family income?

   .... (THB)

5. How long have you been stay in this province?

   .... (years)

6. What is your ethnicity?

   ☐ 1. Burma

   ☐ 2. Others

7. What is your highest education?

☐ 1. Primary school or lower

☐ 2. Above primary school

8. Do you have any current occupation?

☐ 1. Yes

☐ 2. No

9. Have you ever smoke?

☐ 1. Current

☐ 2. Not current

☐ 3. Never

10. Did you have any contact to others' smoking during the present pregnancy?

☐ 1. Yes

☐ 2. No

11. Did you take any aerobic exercise (i.e. walking about 20 minutes per day) during this pregnancy?

☐ 1. Yes

☐ 2. No
