# Supplementary material for: Blood heavy metals and brain-derived neurotrophic factor in the first trimester of pregnancy among migrant workers
Source: PLoS One. 2019 Jun 14;14(6):e0218409. doi: 10.1371/journal.pone.0218409 (PMC6570031; doi:10.1371/journal.pone.0218409)
Supplement: S3 File — (PDF) [file pone.0218409.s003.pdf]

# ကိုယ်တိုင်ဖတ်ပြီးဖြေဆိုရသော မေးခွန်းလွှာ

ဤသုတေသနသည် ထိုင်းနိုင်ငံရှိ ရွှေ့ပြောင်းအလုပ်သမားများ၏ ကိုယ်ဝန်ပထမ၃လအတွင်း မိခင်သွေးထဲမှ သတ္တုလေးများနှင့် အာရုံကြော ဖွံ့ဖြိုးစေရေးအထောက်အပံ့ပေးပရိုတင်းတို့အား လေ့လာခြင်းဖြစ်ပါသည်။ မေးခွန်းများအား ကိုယ်တိုင်ဖတ်ပြီးဖြေပေးပါရန် လေးစားစွာ ဖိတ်ခေါ်အပ်ပါသည်။ သင်ရွေးသည့် အဖြေဘေးရှိအကွက်ထဲတွင် ကြက်ခြေခတ်သင်္ကေတ ထည့်ခြင်းဖြင့် အဖြေမှန်များကို ရွေးချယ်ဖြေဆိုပါ။ မေးခွန်းများတွင် သင်၏လူမှုရေးရာအခြေခံအချက်များနှင့် ကျန်းမာရေးအမူအကျင့်များ ပါဝင်မည်ဖြစ်ပါသည်။ မေးခွန်းများဖြေဆိုရာတွင် ဆက်လက်ဖြေဆိုရန်ဆန္ဒမရှိတော့ပါက လွတ်လပ်စွာ ငြင်းပယ်ခွင့်ရှိပါသည်။ (သို့မဟုတ်) မဖြေဆိုလိုသည့် မေးခွန်းများအား ကျော်သွားနိုင်ပါသည်။ သင့်အဖြေများကို စနစ်တကျလုံခြုံစွာ ထိန်းသိမ်းပေးသွားမည်ဖြစ်ပြီး သင်ဟု သိစေနိုင်မည့် အမည်၊ နေရပ်လိပ်စာနှင့် အခြားမည်သည့်အချက်အလက်ကိုမျှ ဖော်ပြသွားမည် မဟုတ်ပါ။

၁။ အသက်

................. (နှစ်)

၂။ ကိုယ်အလေးချိန်

................. (ကီလိုဂရမ်)

၃။ အရပ်အမြင့်

................. (စင်တီမီတာ)

၄။ သင့်မိသားစုတစ်လဝင်ငွေမှာ ခန့်မှန်းမည်မျှရှိပါသလဲ။

................. (ထိုင်းဘတ်)

၅။ ဤခရိုင်၊ မြို့နယ်အတွင်းနေထိုင်သည်မှာ မည်မျှကြာပြီလဲ။

....................... (နှစ်)

၆။ သင်ဘာလူမျိုးလဲ။

- ☐ ၁။ ဗမာ
- ☐ ၂။ အခြားလူမျိုး

၇။ အတန်းပညာမည်မျှအထိ ပြီးမြောက်ခဲ့ပါသလဲ။

☐ ၁။ မူလတန်းနှင့် မူလတန်းအောက်

☐ ၂။ မူလတန်းအထက်

၈။ လက်ရှိအလုပ်အကိုင် ရှိပါသလား။

☐ ၁။ ရှိပါတယ်

☐ ၂။ မရှိပါ

၉။ ဆေးလိပ်သောက်ဖူးပါသလား

☐ ၁။ လတ်တလောသောက်ပါတယ်

☐ ၂။ အခုမသောက်ပါ၊ အရင်ကသောက်ဖူးပါတယ်

☐ ၃။ လုံးဝမသောက်ဖူးပါ

၁၀။ ယခုကိုယ်ဝန်ဆောင်ကာလအတွင်းဆေးလိပ်ငွေ့ရှူမိတာ၊အခြားသူများ

ဆေးလိပ်သောက်သည့်အနား နေဖူးတာ ရှိပါသလား။

☐ ၁။ ရှိပါတယ်

☐ ၂။ မရှိပါ

၁၁။ ယခုကိုယ်ဝန်ဆောင်ကာလအတွင်း လေ့ကျင့်ခန်း (နေ့စဉ်၂ဂမိနစ်ခန့် လမ်းလျှောက်ခြင်း) လုပ်ဖြစ်ပါသလား။

- [ ] ၁။ လုပ်ဖြစ်ပါတယ်
- [ ] ၂။ မလုပ်ဖြစ်ပါ
